# Knowledge of and adherence to radiographic guidelines for low back pain: a survey of chiropractors in Newfoundland and Labrador, Canada

**DOI:** 10.1186/s12998-020-00361-2

**Published:** 2021-01-18

**Authors:** Diana De Carvalho, André Bussières, Simon D. French, Darrell Wade, Debbie Brake-Patten, Lino O’Keefe, Barbara Elliott, Ken Budgell, Sara O’Reilly, Daphne To, Amanda Hall

**Affiliations:** 1 Division of Community Health and Humanities, Faculty of Medicine, Memorial University of Newfoundland, St. John’s, NL A1B 3V6 Canada; 2 School of Physical and Occupational Therapy, McGill University, Montréal, QC Canada; 3 Département chiropratique, Université du Québec à Trois-Rivières, Trois-Rivières, QC Canada; 4 Department of Chiropractic, Macquarie University, Sydney, New South Wales Australia; 5 Private Practice, St. John’s, NL Canada; 6 Private Practice, Bay St. George, NL Canada; 7 Private Practice, Terra Nova, NL Canada; 8 Patient Engagement Partner, North Bay, ON Canada; 9 Faculty of Education and Professional Studies – School of Nursing, Nipissing University, North Bay, ON Canada; 10 Primary Healthcare Research Unit, Faculty of Medicine, Memorial University of Newfoundland, St. John’s, NL Canada

**Keywords:** Diagnostic imaging, Low back pain, Guidelines, Knowledge and beliefs, Chiropractors

## Abstract

**Background:**

Low back pain (LBP) rarely requires routine imaging of the lumbar spine in the primary care setting, as serious spinal pathology is rare. Despite evidence-based clinical practice guidelines recommending delaying imaging in the absence of red flags, chiropractors commonly order imaging outside of these guidelines. The purpose of this study was to survey chiropractors to determine the level of knowledge, adherence to, and beliefs about, clinical practice guidelines related to the use of lumbar radiography for LBP in Newfoundland and Labrador (NL), Canada.

**Methods:**

A cross-sectional survey of chiropractors in NL (*n* = 69) was conducted between May and June 2018, including questions on demographics, awareness of radiographic guidelines, and beliefs about radiographs for LBP. We assessed behavioural simulation using clinical vignettes to determine levels of adherence to LBP guideline recommendations.

**Results:**

The response rate was 77% (*n* = 53). Half of the participants stated they were aware of current radiographic guideline recommendations, and one quarter of participants indicated they did not use guidelines to inform clinical decisions. The majority of participants agreed that x-rays of the lumbar spine are useful for patients with suspected pathology, are indicated when a patient is non-responsive to 4 weeks of conservative treatment for LBP, and when there are neurological signs associated with LBP. However, a small proportion indicated that there is a role for full spine x-rays (~ 21%), x-rays to evaluate patients with acute LBP (~ 13%), and that patient expectations play a role in decision making (4%). Adherence rate to radiographic guidelines measured using clinical vignettes was 75%.

**Conclusions:**

While many chiropractors in this sample reported being unsure of specific radiographic guidelines, the majority of respondents adhered to guideline recommendations measured using clinical vignettes. Nonetheless, a small proportion still hold beliefs about radiographs for LBP that are discordant with current radiographic guidelines. Future research should aim to determine barriers to guideline uptake in this population in order to design and evaluate tailored knowledge translation strategies to reduce unnecessary LBP imaging.

**Supplementary Information:**

The online version contains supplementary material available at 10.1186/s12998-020-00361-2.

## Introduction

Lumbar radiography plays an important role in the management of low back pain (LBP) when pathology (tumor, infection, or inflammatory arthropathy) or trauma (fracture or dislocation) are suspected [1]. However, these LBP causes are extremely rare, comprising approximately 5% of cases [2]. Strong evidence indicates that routine imaging of the spine does not improve patient outcomes, increases exposure to unnecessary harms, and increases costs [3, 4]. Accordingly, guidelines in both the fields of medicine [5] and chiropractic [6] recommend delaying imaging, in the absence of red flags, for 4 to 6 weeks. If there is no response to conservative care after this period, then imaging may be indicated [3, 6]. Despite this, a large proportion of clinicians request lumbar imaging outside of these guideline recommendations [7, 8].

In the field of chiropractic, several survey-based studies have found lumbar spine radiography utilization rates vary widely worldwide from 25 to 93% [9–14]. In Canada, a 1997 study found utilization rates of 63–68% in an Ontario community with a large proportion for reasons outside the recommendations of clinical guidelines [15], self-reported utilization rates by interviewed chiropractors in Ontario and Quebec were 10–50% in 2010 [16], while rates of lumbar spine radiographs funded by the Ontario Health Insurance Plan and Workplace Safety and Insurance Board in 2000/2001 were estimated at 3.25 and 3.30% respectively [17]. Unsupported reasons for ordering lumbar films include: to screen for congenital abnormalities and contraindications to spinal manipulative therapy [13], biomechanical/postural analysis of the spine and to educate the patient [15], and medicolegal reasons [16].

Current utilization rate of lumbar radiography for LBP by chiropractors in the province of Newfoundland and Labrador (NL) is unknown. From informal data tracked by the Newfoundland and Labrador Chiropractic Board, it is estimated that the rate could be as high as 36% (based on data from the Avalon Peninsula for 2015 assuming 2 new patients/week). The awareness of current radiographic guidelines, the uptake of those guidelines, and beliefs towards plain film radiography for patients with LBP has not been previously studied in this clinician population. Therefore, the purpose of this study was to survey chiropractors to determine the level of knowledge, adherence to, and beliefs about, clinical practice guidelines related to the use of lumbar radiography for LBP in NL, Canada. A secondary objective was to estimate level of adherence to radiographic guidelines using clinical vignette responses (i.e., behaviour simulation), and to compare our findings to published estimates by Walker et al. (2011) [18] in Australia.

## Methods

This study used a cross-sectional design. All chiropractors registered in the province of NL (*n* = 69) were invited to participate in an anonymous online survey from May to June 2018. The initial survey invitation, link to the survey, and reminders were sent from the Newfoundland and Labrador Chiropractic Association directly. The survey window was open for a period of 6 weeks, with reminder emails sent out weekly. The Newfoundland and Labrador Health Research Ethics Authority granted ethics approval prior to the start of this study (#20181407). Participants read an information letter at the start of the survey and clicking to start the survey implied consent to participate. If participants wished, they could additionally consent to being entered into a draw for a tablet computer. Survey responses and draw entries were not linked. Participants who had not been in practice (involved in direct patient care) for greater than 1 year were excluded (survey ended after question 2).

### Survey

Survey questions were adapted from prior studies administered to chiropractors in Australia by Jenkins et al. (2016) [12] and Walker et al. (2011) [18]. Specifically, the survey was comprised of questions from Jenkins et al. (2016) [12] with minor changes to address regional differences in language or practice, to gather additional information and/or present additional radiographic guideline options. Specific differences between the survey tools, and the survey in its entirety, have been included in supplementary material 1. The survey had four sections: demographics, awareness of radiographic guidelines, beliefs about radiographs for LBP, and adherence to guidelines. Specifically, there were seven demographic questions asking participants for the chiropractic college they attended, year of graduation, years of practice, workload, practice setting (urban vs. rural), and technique system used.

Two questions inquired about participants’ awareness of published guidelines: ‘Whether they were aware of current radiographic guidelines for LBP’ (response options were a yes/no/unsure), and ‘Which guidelines they were familiar with’ (from a list provided) or whether ‘they did not use guidelines to inform decisions’. A comment box was included to report guidelines not listed.

Three questions, including several statements related to their beliefs about lumbar spine radiographs for LBP, asked participants to rate their level of agreement with each statement on a five point scale (from “strongly disagree” to “strongly agree”).

Finally, behavioural simulation used responses to five written clinical vignettes to assess participants’ level of adherence to LBP guideline recommendations on the use of lumbar spine radiography. The vignettes were previously used in a study by Walker et al. in 2011 and were designed to reflect patients with acute LBP who would typically present to chiropractors [18]. The vignettes were constructed based on recommendations from a diagnostic imaging guideline [19], key elements that may influence chiropractors’ decisions to manage uncomplicated back pain without lumbar spine x-rays that were previously identified in the literature [16, 20, 21], and through expert opinion of the clinical members of the research team. The vignettes were presented exactly as they were given in Walker et al. (2011) [18] with the exception of referring to a “heated wheat pack” as a “hot pack”. The full vignettes can be found in the supplementary material of Walker et al. (2011) [18]. For each scenario, participants were asked to indicate whether they would recommend lumbosacral plain film x-ray, full spine x-rays, or no x-rays, and to expand on their clinical decision using a comment box (no word limit). The complete survey is available in the supplemental data section.

### Statistical analysis

We conducted a descriptive analysis (proportions and 95% confidence intervals) of the demographic, awareness of radiographic guidelines, and beliefs questions. Exact binomial confidence intervals were calculated for proportions that were small. Due to the small population of chiropractors in the province, paired with the need to protect anonymity of those participating in the survey, logistical regression to chiropractor characteristics was not performed. Missing responses were tracked and reported. All proportions were calculated based on the total number of respondents, excluding those who were not involved in direct patient care for longer than 1 year.

A 2 × 2 contingency table (Table 1) with expected (according to guideline recommendations) versus observed (survey results) recommendations was then used to calculate adherence to guidelines for each of the five vignettes. Since the clinical vignettes used in this study were essentially identical to those used in Walker et al. (2011) [18], and given the similarities in clinical guidelines internationally should ideally translate to similar practice, proportions of recommendations for lumbosacral x-ray, full spine x-ray, or no recommended x-ray were then compared with those from the original study using a N-1 Chi-squared test with statistical significance accepted at *p* < 0.05.

**Table 1:** A 2 × 2 contingency table outlining how adherence to guidelines was calculated. Adherence (no x-ray) was calculated as the percentage of cases where radiography was not recommended by survey participants among all cases where radiographs were not indicated according to guidelines (d/(c + d)) × 100%. Similarly, adherence (x-ray) was calculated as the percentage of cases where radiography was recommended by survey participants among all cases where radiographs were indicated according to guidelines (a/(a + b)) × 100%

		Survey Recommendation		
		Yes	No	Total
**Radiographs indicated according to guidelines**	**Yes**	a	b	a + b
	**No**	c	d	c + d
**Total**		a + c	b + d	

## Results

### Demographics

The survey response rate was 77% (*n* = 53). Table 2 details the characteristics of the population surveyed. We excluded responses from one participant who identified as not being involved in direct patient care for longer than 1 year.

**Table 2:** Demographic characteristics of the survey participants (*n* = 52) presented as percentages (95% confidence intervals)

Variables	Categories	Number of Participants n^**a**^ (%; 95% CI)
Currently in practice (i.e. involved in direct patient care)	Yes	51/53 (96.2%; 91.1–100%)
	No, on leave	1/53 (1.9%; 0–10%)^b^
	No, have not practiced for > 1 year^c^	1/53 (1.9%; 0–10%)^b^
Workload	Full Time	46 (88.46%; 79.8–97.2%)
	Part Time (≤ 12 h/week and/or ≤ 60 patients/week)	6 (11.54%; 2.9–20.2%)
Practice Setting	Urban	39 (75%; 63.2–86.8%)
	Rural	12 (23.08%; 11.6–34.5%)
	Both	1 (1.92%; 0–10.3%)^b^
Chiropractic School Attended	Canadian Memorial Chiropractic College	30 (57.69%; 44.3–71.1%)
	Central Queensland University	1 (1.92%; 0–10.3%)^b^
	Cleveland Chiropractic College	4 (7.69%; 0–18.5%)^b^
	Logan University	1 (1.92%; 0–10.3%)^b^
	Palmer College of Chiropractic	15 (28.85%; 16.4–41.2%)
Practice Years	0 to 12	21 (40.38%; 27.1–53.7%)
	13 to 24	23 (44.23%; 30.7–57.7%)
	25 to 37	7 (13.46%; 4.2–22.7%)
Technique Used	Diversified	45 (86.54%; 77.3–95.8%)
	Gonstead	2 (3.85%; 0.5–13.2%)^b^
	Thompson Technique	1 (1.92%; 0–10.3%)^b^
	Sacrooccipital Technique	1 (1.92%; 0–10.3%)^b^
	Leander Flexion Distraction	1 (1.92%; 0–10.3%)^b^
Aware of Current Radiographic Guidelines for LBP?	Yes	26 (50%; 36.4–63.6%)
	No	5 (9.62%; 1.6–17.6%)
	Unsure	18 (34.62%; 21.7–47.6%)
Continuing Education in Radiographic Indications/Guidelines	Yes	14 (26.92%; 14.9–39%)
	No	36 (69.23%; 56.7–81.8%)

^a^Total number of participants (denominator) = 52 unless otherwise specified

^b^Exact binomial confidence interval calculated

^c^Survey ended after the first question

### Awareness of radiographic guidelines

Half of the participants (26/52, 50%, 95% CI 36%; 64%) stated they were aware of current radiographic guidelines for LBP, five participants were not (5/52, 10%, 95% CI 2%; 18%), and several others were unsure (18/52, 35%, 95% CI 22%; 48%); three participants did not respond to this question (Table 2).

**Fig. 1:**
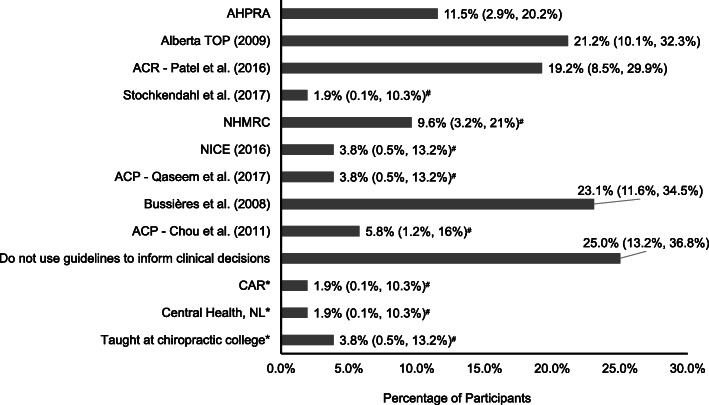
Percentage of participants (95% confidence interval) that were aware of the following radiographic guidelines for low back pain: Australian Health Practitioner Regulation Agency (AHPRA) [24, 25]; Alberta Toward Optimized Practice (TOP) Evidence-informed primary care management of low back pain: Clinical practice guideline [22]; American College of Radiology (ACR) Appropriateness criteria for low back pain Patel et al. [23]; National Clinical Guidelines for non-surgical treatment of patients with recent onset low back pain or lumbar radiculopathy Stochkendahl et al. [26], National Health and Medical Research Council (NHMRC) Evidence-based management of acute musculoskeletal pain [19]; National Institute for Health and Care Excellence (NICE) Low back pain and sciatica in over 16 s: assessment and management [27]; American College of Physicians (ACP) Noninvasive treatments for acute, subacute, and chronic low back pain Qaseem et al. [28]; Diagnostic imaging practice guidelines for musculoskeletal complaints in adults-an evidence-based approach-part 3: spinal disorders Bussières et al. [6]; ACP Diagnostic imaging for low back pain: Advice for high-value health care Chou et al. [29]; CAR: Canadian Association of Radiologists; NL: Newfoundland and Labrador. (^*^Entered by participants in the survey in response to “other”; ^#^Exact binomial confidence intervals calculated)

A similar proportion of participants reported they were familiar with the Alberta LBP guidelines [22] (11/52, 21%, 95% CI 10%; 32%), the American College of Radiology guidelines [23] (10/52, 19%, 95% CI 9%; 30%), and the Diagnostic Imaging Practice Guidelines for Musculoskeletal Complaints in Adults [6] (12/52, 23%, 95% CI 12%; 35%). One quarter of participants (13/52, 25%, 95% CI 13%; 37%) indicated that they do not use guidelines to inform their clinical decisions; nine participants did not respond to this question (Fig. 1). Three other guidelines were provided by participants: the Canadian Association of Radiologists Guidelines, Central Health Guidelines (local health authority), and what was taught at their chiropractic college.

### Beliefs about lumbar spine imaging

**Fig. 2:**
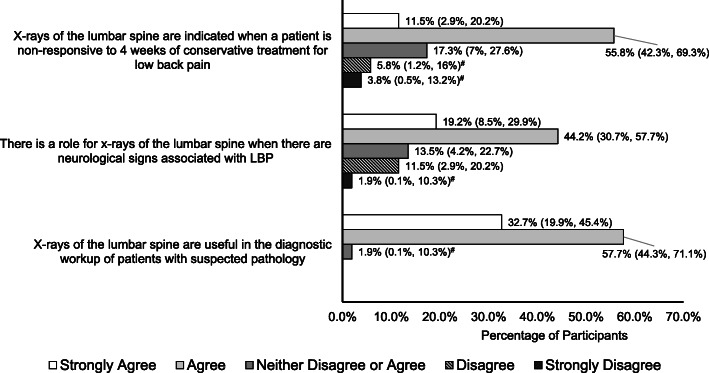
Statements that majority of participants agreed (agreed or strongly agreed) to. Proportions (95% confidence interval) of participants agreeing or disagreeing with statements that probe beliefs about radiographic imaging for low back pain. Note, the count does not add up to 100% due to missing responses. (^#^Exact binomial confidence intervals calculated)

**Fig. 3:**
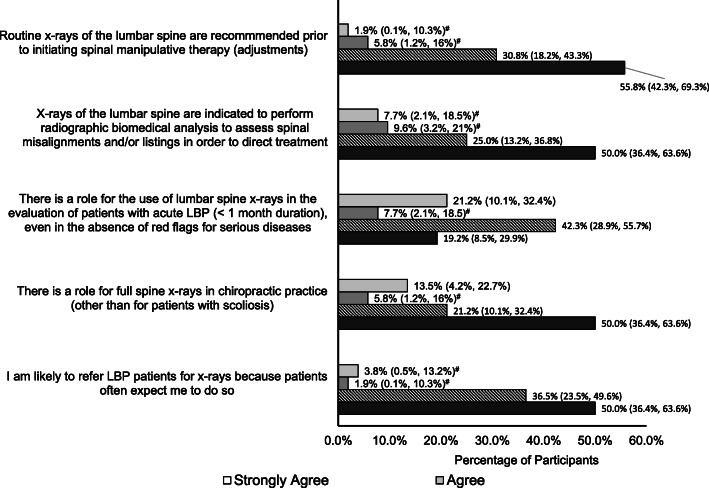
Statements that majority of participants disagreed (disagreed or strongly disagreed) to. Proportions (95% confidence interval) of participants agreeing or disagreeing with statements that probe beliefs about radiographic imaging for low back pain. Note, the count does not add up to 100% due to missing responses. (^#^Exact binomial confidence intervals calculated)

**Fig. 4:**
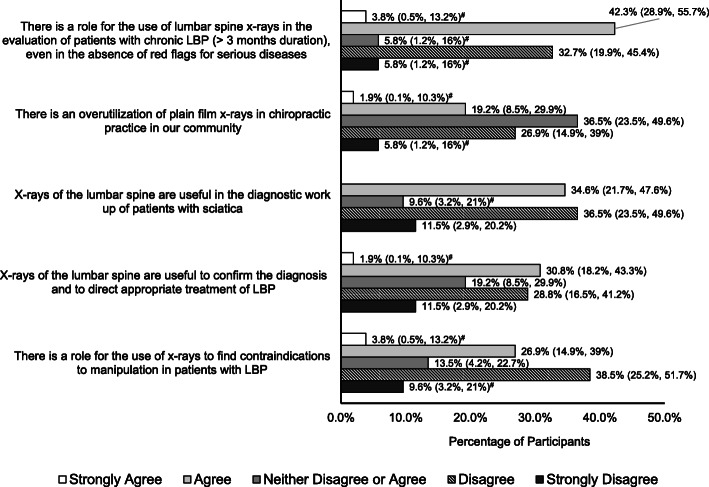
Statements with less unified beliefs (no majority). Proportions (95% confidence interval) of participants agreeing or disagreeing with statements that probe beliefs about radiographic imaging for low back pain. Note, the count does not add up to 100% due to missing responses. (^#^Exact binomial confidence intervals calculated)

Beliefs about radiographic imaging for LBP from the series of questions adapted from Jenkins et al. (2016) [12] are reported in Figs. 2, 3 and 4.

In summary, the majority of participants agreed (Agree or Strongly Agree) that (Fig. 2):

- X-rays of the lumbar spine are indicated when a patient is non-responsive to 4 weeks of conservative treatment for LBP (67%, 35/52, 3 missing responses).
- There is a role for x-rays of the lumbar spine when there are neurological signs associated with LBP (64%, 33/52, 5 missing responses).
- X-rays of the lumbar spine are useful in the diagnostic workup of patients with suspected pathology (90%, 47/52, 4 missing responses).

In contrast, the majority of participants disagreed (Disagree or Strongly Disagree) that (Fig. 3):

- Routine x-rays of the lumbar spine are recommended prior to initiating spinal manipulative therapy (87%, 45/52, 3 missing responses).
- X-rays of the lumbar spine are indicated to perform radiographic biomedical analysis to assess spinal misalignments and/or listings in order to direct treatment (75%, 39/52, 4 missing responses).
- There is a role for the use of lumbar spine x-rays in the evaluation of patients with acute LBP (< 1 month duration), even in the absence of red flags for serious disease (62%, 32/52, 5 missing responses).
- There is a role for full spine x-rays in chiropractic practice (other than for patients with scoliosis) (71%, 37/52, 5 missing responses).
- They would be likely to refer LBP patients for x-ray because patients often expect them to do so (87%, 45/52, 4 missing responses).

Statements that demonstrated less unified beliefs (no majority) included (Fig. 4):

- There is a role for the use of lumbar spine x-rays in the evaluation of patients with chronic LBP (> 3 months duration), even in the absence of red flags for serious diseases (39%, 20/52 disagreed; 6%, 3/52 neither disagreed or agreed; 46%, 24/52 agreed; 5 missing responses).
- There is an overutilization of plain film x-rays in chiropractic practice in our community (33%, 17/52 disagreed; 37%, 19/52 neither disagreed or agreed; 21%, 11/52 agreed; 5 missing responses).
- X-rays of the lumbar spine are useful in the diagnostic work up of patients with sciatica (48%, 25/52 disagreed; 10%, 5/52 neither disagreed or agreed; and 35%, 18/52 agreed; 4 missing responses).
- X-rays of the lumbar spine are useful to confirm the diagnosis and to direct appropriate treatment of LBP (40%, 21/52 disagreed; 19%, 10/52 neither disagreed or agreed; 33%, 17/52 agreed; 4 missing responses).
- There is a role for the use of x-rays to find contraindications to manipulation in patients with LBP (48%, 25/52 disagreed; 14%, 7/52 neither disagreed or agreed; 31%, 16/52 agreed; 4 missing responses).

### Adherence based on clinical scenarios

Adherence (no x-ray) to radiographic guidelines, where no x-ray was chosen when not indicated by guidelines, was estimated at 75%. Adherence (x-ray) to radiographic guidelines, where an x-ray was chosen when it was indicated by guidelines, was estimated at 91%. For the clinical vignette where imaging was indicated according to guideline recommendations (Vignette 5), most participants (77%, *n* = 40) stated they would order imaging. For the four other vignettes where imaging was not indicated, the proportion of practitioners responding “no imaging recommended” in adherence to guideline recommendations ranged from 88% (*n* = 46, Vignette 1), 75% (*n* = 39, Vignette 2), 38% (*n* = 20, Vignette 3), and 62% (*n* = 32, Vignette 4).

Since the clinical vignettes used in this study were adapted from Walker et al. (2011) [18], a comparison of proportional responses for “lumbosacral x-ray”, “full spine x-ray”, and “none” for each scenario was calculated (Table 3). For all scenarios except vignette 3, a significantly larger proportion of participants in the current study indicated a recommendation that was in accordance to radiographic guidelines. Further, across all scenarios, a significantly lower proportion of participants in the current study indicated that full spine x-rays would be warranted.

**Table 3:** A summary of responses (proportion (95% confidence interval)) for each of the five clinical vignettes from the current study (top) compared with the corresponding results from the Walker et al. (2011) [18] study (proportion differences (95% confidence interval)) (bottom)

Recommendation	Vignette 1 Acute LBP without radiculopathy Non-traumatic	Vignette 2 Chronic LBP without radiculopathy	Vignette 3 Subacute LBP without radiculopathy	Vignette 4 Acute LBP without radiculopathy Non-traumatic	Vignette 5 Acute LBP Traumatic
**Lumbosacral X-ray**	2% (0–1%)^f^	12% (3–20%)	46% (33–60%)	23% (12–35%)	71% (59–83%)
**Full Spine X-ray**	0%	2% (0–10%)^f^	0%	2% (0–10%)^f^	6% (1–16%)^f^
**None**	88% (80–97%)	75% (63–87%)	38% (25–52%)	62% (48–75%)	8% (0–15%)
**Difference from Walker et al. (2011)** [18]**, compared by the N-1 Chi-Squared test**					
**Lumbosacral X-ray**	↓ 38% (28–44%)^a^	↓ 40% (27–49%)^a^	1% (−13–15%)	↓ 17% (3–28%)^b^	↑ 4% (−10–16%)
**Full Spine X-ray**	↓ 24% (16–29%)^a^	↓ 30% (20–36%)^a^	↓ 14% (6–19%)	↓ 23% (13–29%)^c^	↓ 22% (11–29%)^d^
**None**	↑ 54% (41–62%)^a^	↑ 61% (47–71%)^a^	↑ 18% (5–32%)^e^	↑ 34% (19–47%)^a^	5% (− 0.5–16%)

Significance at *p*< 0.05. ^a^ *p* < 0.0001, ^b^*p* = 0.0203, ^c^*p* = 0.0002, ^d^*p* = 0.0007, ^e^*p* = 0.0041

^f^Exact binomial confidence interval calculated

Arrows indicate direction of proportion (↓ lower, ↑ higher) compared to Walker et al. (2011) [18]. The only clinical vignette where recommending a lumbar spine x-ray would adhere to the current guidelines was #5

## Discussion

Chiropractors in NL have comparable levels of knowledge of radiographic guidelines to a previously published study in Australia [12]; with approximately 50% of the participants being aware of at least one guideline for lumbar spine radiography. Twenty-five percent of participants indicated that they do not use guidelines to inform clinical decisions and 4% indicated that they were relying on information presented to them when in chiropractic college. Together, these results suggest that interventions aimed at improving awareness and uptake of clinical guidelines, and ultimately improve quality of care, may be warranted.

The majority of beliefs held by participants regarding the use of lumbar spine x-rays for LBP suggest that chiropractors in NL are generally well informed about LBP and its management. The results of this section of the survey are consistent with those from the original study conducted by Jenkins et al. (2016) [12], with a few notable exceptions. These included a lower proportion of respondents indicating a role for x-rays for LBP when neurological signs are present, for routine x-rays prior to initiating SMT, in acute LBP, in patients with sciatica, or to confirm diagnosis and to direct treatment. However, a higher proportion of respondents agreed that they would refer LBP patients for x-ray because patients expect them to. These differences may be due to the fact that chiropractors in NL are mandated to complete continuing education in the area of radiology each year in order to maintain licensure. However, given that Jenkins et al. (2016) [12] demonstrated that chiropractors taking their own x-rays, who are practicing techniques other than diversified, or are unaware of current radiographic guidelines were associated with poorer adherence, it may be due to the fact that this population does not have access to in-house radiographic facilities and mainly (87%) practice diversified.

Despite larger proportions demonstrating beliefs that are generally in line with current recommendations, not all practitioners are clear on the role of lumbar spine x-rays for sciatica, chronic LBP, in the general management of LBP, and for the determination of spinal manipulative therapy contraindications. Therefore, these topics do need to be revisited more frequently throughout the mandated continuing education program. For example, the survey identified a few areas for further consideration. First, 21% of participants agreed that there is a role for the use of lumbar spine x-rays in the evaluation of patients with acute LBP (< 1-month duration) in the absence of red flags for serious disease, despite strong evidence recommending against imaging in this case. Secondly, 13% believed that full spine x-rays (other than for patients with scoliosis) have a role to play in chiropractic practice when the diagnostic value from full spine x-rays is known to be poor. Finally, while the majority of practitioners indicated that they were not likely to refer LBP patients for x-ray because patients often expect them to do so (87%, 45/52), 4% of the population indicated that patient expectations do play a role in their decision making. These results suggest there is room for improvement to enhance patient care. Evidence based approaches to address these concerns include the use of shared decision aids [30], aimed at both chiropractors and their patients, and clinician decision supports, such as modified referral forms allowing for guideline-appropriate indications for imaging and targeted reminders such as short educational messages promoting correct imaging practices [31].

While not a perfect representation of actual clinical practice [32], the responses to the vignettes suggest that the adherence to radiographic guidelines is fairly high (75% for not ordering an x-ray when it is not indicated by guidelines and 91% for ordering an x-ray when it is indicated by guidelines) for many cases typical of practice, and that these levels are significantly greater than those reported previously by Walker et al. (2011) [18]. This does suggest that there is room to improve the adherence to guidelines for the reduction of imaging where it is not recommended (low-value care). These results also suggest that there does not appear to be an underuse of imaging when it would be necessary, which also could be a problem, as discussed by Jenkins et al. (2018) [7]. Finally, the increased proportion of respondents indicating they would order imaging in Vignette 3, which included contextual factors of a busy clinic and a frustrated/unhappy patient, suggests that patient expectations may play a role in decision making. This is not unexpected and is consistent with the literature [33], however, it is contradictory to the result of the belief statement in this study (87% indicated patient expectations do not play a role) suggesting that further education and training on how to handle these situations is warranted.

The strong disagreement regarding the belief that full spine x-rays play a role in the management of LBP (excluding scoliosis) was reflected in lower proportions of participants in this study recommending full spine views compared to the results of Walker et al. (2011) [18]. There may be a few reasons for the difference in adherence. First, this study was conducted approximately 10 years after the study by Walker et al. [18], and beliefs about the role of x-rays for the management of LBP may have changed since then. Second, in Newfoundland and Labrador all radiographs are taken in public healthcare facilities (i.e. hospitals) and true “full spine” (14″× 51″ cassette) are not taken. When full spine requests are ordered, separate cervical, thoracic and lumbar series are done instead which may be different enough from the single view that practitioners do not order them as frequently. Finally, we are comparing populations from different countries. However, we would expect that since international guidelines around LBP and imaging are similar enough, we should see similar behaviours in these groups, thus justifying the comparison of these populations.

There was a large degree of uniformity across responses particularly in the beliefs and clinical vignettes sections. This uniformity may be due to the practitioner population being in practice for less than 24 years (12 years or less (40%) and between 13 and 24 years (44%), the high proportions of the population primarily using Diversified Technique (86%), a non-x-ray driven chiropractic technique, and the majority being trained by the same institution (58% graduated from the Canadian Memorial Chiropractic College). Anecdotally, it has been suggested that the practitioners in the province of NL have a strong sense of community and generally have similar practice styles. Whether or not this local culture has had an effect on these results is unknown; however, it may be worth investigating in the future.

### Strengths and limitations

This study had a high response rate. However, results from cross-sectional surveys are still limited by sampling error (random differences between the sample and the total population), nonresponse error (error introduced by the proportion of the population that did not complete the survey), recall bias (error introduced by faulty memory), and measurement error (i.e. errors in reading a question, misrepresenting the truth). We have only represented awareness of guidelines by recognition of given citations, which may have overestimated whether clinicians are indeed knowledgeable of these particular documents. Clinical vignettes are not an ideal representation of what happens in actual clinical practice; however, they have been shown to be a valid way of assessing the quality of practitioner practice [34]. While we were able to compare our results to those from Walker et al. (2011) [18], there is a chance that some of the participants may have been familiar with these scenarios from the literature, which would have biased the results.

## Conclusion

Chiropractors in Newfoundland and Labrador appear to be a relatively homogenous group in terms of years of practice, education, and clinical approach, and they appear to have fairly unified beliefs regarding the use of lumbar spine x-ray for LBP. While the majority of practitioners in this province appear to follow the current recommendations for lumbar spine radiography in patients with LBP, areas for improvement have been identified. Future research should aim to determine factors hindering guideline awareness and adherence in this population and evaluate the impact of tailored knowledge translation strategies to reduce unnecessary LBP imaging.

## Supplementary Information

**Additional file 1.**

## Data Availability

All data generated or analysed during this study are included in this published article and its supplementary information files.
