# Supplementary material for: Knowledge of and adherence to radiographic guidelines for low back pain: a survey of chiropractors in Newfoundland and Labrador, Canada
Source: Chiropr Man Therap. 2021 Jan 18;29:4. doi: 10.1186/s12998-020-00361-2 (PMC7812732; doi:10.1186/s12998-020-00361-2)
Supplement: Supplementary file 1 — Additional file 1. [file 12998_2020_361_MOESM1_ESM.zip › 12998_2020_361_MOESM1_ESM.pdf]

Welcome

Researchers from Memorial University, McGill University, Queen's University, and the Newfoundland and Labrador Chiropractic Association are collaborating to conduct a study exploring knowledge of and adherence to radiographic guidelines for low back pain. The study is funded by NL Support's Translational and Personalized Medicine Initiative. Its purpose is fill a gap in the literature concerning imaging habits of NL chiropractors and help inform the development of a knowledge translation intervention, ultimately contributing to improved patient health.

We invite you to complete the following survey, which should take about 15 - 20 minutes. Your input may help us better understand knowledge of and adherence to radiographic guidelines for low back pain among chiropractors in NL.

This anonymous survey includes questions about your practice, your radiographic requisition practice, and your knowledge of existing guidelines. We also ask for some demographic information. Note that your name will not be linked to your responses, and any potentially identifying information will be kept confidential. You will receive instructions at the end of the survey for an opportunity to enter a draw for an iPad. Your contact information will NOT be linked to your survey responses.

Survey data will be stored at Memorial University behind firewalls on a secure server with a password-protected login. The data will be kept for a period of 5 years.

Thank you for your time in this effort, we do look forward to hearing from you. If you would like to discuss this study in more detail please contact the study’s Principal Investigator, Dr. Diana De Carvalho (contact information below). If you have questions regarding your rights as a research participant, here is the contact information for the Health Research Ethics Office: 709-777-8776 or info@hrea.ca.

Thank you,

Diana De Carvalho DC, MSc, PhD Memorial University of Newfoundland
Diana.DeCarvalho@med.mun.ca
709-777-8955

Darrell Wade, DC
NL Chiropractic Association
nlca@nl.rogers.com
709-739-7762

* 1. Do you wish to participate in this survey and consent to having these data collected? (Please note all data collection is completely anonymous).

- ◯ Yes
- ◯ No

Chiropractor and chiropractor practice characteristics

* 2. Are you currently practicing? (i.e., involved in direct patient care)

- ◯ Yes
- ◯ No, currently on leave less than or equal to one year
- ◯ No, have been away from practice for greater than one year

Work load

3. How would you describe your current work load in private practice?

- ◯ Full-time
- ◯ Part-time (12 hours or less per week and/or treating fewer than 60 patients per week)

Practice characteristics

4. Do you predominantly practice in a rural or an urban setting?

- ◯ Urban setting
- ◯ Rural setting
- ◯ I practice in both rural and urban settings

5. At which institution did you complete your chiropractic studies?

6. What year did you finish your chiropractic studies?

7. How many years have you been in practice?

Practice techniques

8. Which chiropractic technique or system do you **mainly** utilize?

- ◯ Diversified
- ◯ Gonstead
- ◯ Activator methods
- ◯ Thompson technique
- ◯ Sacrooccipital technique (SOT)
- ◯ Applied Kinesiology (AK)
- ◯ Chiropractic BioPhysics (CBP)
- ◯ Advanced Biostructural Correction (ABC)
- ◯ Other (please specify)

9. As a chiropractor are you aware of the current radiographic guideline(s) for low back pain?

- ◯ Yes
- ◯ No
- ◯ Unsure

10. As a chiropractor, which radiographic guideline/s for low back pain are you aware of? (Please mark all answers that apply)

- [ ] Chiropractic diagnostic imaging conduct and performance: Chiropractic board of Australia (AHPRA)
- [ ] Alberta low back pain guidelines
- [ ] American College of Radiology guidelines: Patel et al. ACR Appropriateness Criteria Low Back Pain.
- [ ] Stochkendahl et al. National Clinical Guidelines for non-surgical treatment of patients with recent onset low back pain or lumbar radiculopathy. Eur Spine J 2017.
- [ ] Lumbar imaging in acute non-specific low back pain: NHMRC, Australian Government
- [ ] Diagnostic imaging pathways: Department of Health, Western Australia
- [ ] Low back pain and sciatica in over 16s: assessment and management (NICE guidelines)
- [ ] Noninvasive Treatments for Acute, Subacute, and Chronic Low Back Pain: A Clinical Practice Guideline From the American College of Physicians: Qaseem et al. (2017)
- [ ] Diagnostic Imaging Practice Guidelines for Musculoskeletal Complaints in Adults—An Evidence-Based Approach—Part 3: Spinal Disorders: Bussières et al. (2008)
- [ ] Diagnostic Imaging for Low Back Pain: Advice for High-Value Health Care From the American College of Physicians (2011)
- [ ] I do not use guidelines to inform my clinical decisions
- [ ] Other (please specify)

11. Have you undertaken any further education on radiographic indications or guidelines since finishing your chiropractic degree?

- ( ) No

Yes, the training I have done includes...

Agreement scales 1/3

The next few pages contain statements about chiropractic practice. Please indicate how well you agree with the statements.

12. Please select a response that indicates how well you agree with the following statements about chiropractic practice:

	Strongly Disagree	Disagree	Neither Disagree nor Agree	Agree	Strongly Agree
X-rays of the lumbar spine are indicated when a patient is nonresponsive to 4 weeks of conservative treatment for low back pain	○	○	○	○	○
Routine x-rays of the lumbar spine are recommended prior to initiating spinal manipulative therapy (adjustments)	○	○	○	○	○
X-rays of the lumbar spine are indicated to perform radiographic biomechanical analysis to assess spinal misalignments and/or listings in order to direct treatment	○	○	○	○	○

Agreement scales 2/3

13. Please select a response that indicates how well you agree with the following statements about chiropractic practice:

	Strongly Disagree	Disagree	Neither Disagree nor Agree	Agree	Strongly Agree
There is a role for the use of lumbar spine x-rays in the evaluation of patients with acute low back pain (less than one month duration), even in the absence of red flags for serious disease	○	○	○	○	○
There is a role for the use of lumbar spine x-rays in the evaluation of patients with chronic low back pain (greater than three months duration), even in the absence of red flags for serious disease	○	○	○	○	○
There is a role for full spinal x-rays in chiropractic practice (other than for patients with scoliosis)	○	○	○	○	○
There is overutilisation of plain film x-rays in chiropractic practice in our community	○	○	○	○	○
There is a role for x-rays of the lumbar spine when there are neurological signs associated with low back pain	○	○	○	○	○

Agreement scales 3/3

14. Please select a response that indicates how well you agree with the following statements about chiropractic practice:

	Strongly Disagree	Disagree	Neither Disagree nor Agree	Agree	Strongly Agree
X-rays of the lumbar spine are useful in the diagnostic work up of patients with sciatica	○	○	○	○	○
X-rays of the lumbar spine are useful in the diagnostic work up of patients with suspected pathology	○	○	○	○	○
X-rays of the lumbar spine are useful to confirm the diagnosis and to direct appropriate treatment of low back pain	○	○	○	○	○
There is a role for the use of x-rays as a screening tool to find contraindications to manipulation in patients with low back pain	○	○	○	○	○
I am likely to refer low back pain patients for x-rays of the lumbar spine because patients often expect me to do so	○	○	○	○	○

Vignettes

This section contains five hypothetical vignettes about patients who present to you with acute low-back pain. In the vignettes we have varied a range of features that might influence your management decisions (in regards to investigations you might order and interventions you might recommend or undertake). At the end of each vignette we ask you to indicate what investigation(s) you would order for the patient described in the vignette, and what intervention(s) you would recommend or undertake for this patient.

We are aware that the vignette format means that skills you may normally draw on, such as evaluating non-verbal cues from the patient and performing a physical examination, cannot be a factor in your assessment. Nevertheless, given this understanding, we hope that you address each vignette and answer the questions as best as you can with the information provided. We have left space for you to comment on your decisions, if you wish.

Patient Vignette 1/5

A 48 year old office worker attends your clinic. He is usually very active, playing lots of sport and doing regular exercise (e.g. jogging, gym). He has low-back pain, rated 5 out of 10. The pain started two weeks ago and is located in the low-back region, right sided, no radiation. The pain is relieved by stretching his low-back and using a hot pack. The pain is worse after playing sport, to the point where in the last week he had to stop mid-game during basketball. He has no previous history of low-back pain. The patient thinks that an x-ray is required to “find out what is wrong”, and he is fearful that movement and activity might make the pain worse.

* 15. Which investigations would you order, undertake or recommend for this patient at this visit? Please tick all that apply:

- [ ] Lumbosacral plain x-ray
- [ ] Full spine plain x-ray
- [ ] None

16. Please tell us why you would, or would not, order, undertake or recommend investigations

Patient Vignette 2/5

A 57 year old office worker sees you for low-back pain. She says her pain began 8 weeks ago. There was no specific incident that caused the pain. The pain is located in the lower back region, with no radiation. The pain is a dull ache (3 out of 10), with occasional sharp “twinges” with certain movements. The pain is relieved by heat and a massage from her spouse. She has no history of low-back pain. The patient is overweight (BMI 30), has mild hypertension, and a family history of type 2 diabetes. The patient rarely does any exercise. During the consultation she indicates to you that she is anxious that she may have a serious disease. The patient says “a friend had low-back pain like this and they had an x-ray and it showed that they really had something major wrong with them”. She repeatedly requests an x-ray during the consultation.

* 17. Which investigations would you order, undertake or recommend for this patient at this visit? Please tick all that apply:

- ☐ Lumbosacral plain x-ray
- ☐ Full spine plain x-ray
- ☐ None

18. Please tell us why you would, or would not, order, undertake or recommend investigations

Patient Vignette 3/5

A 36 year old real estate agent consults you for his low-back pain. He comes in on a very busy day at the practice and there are many patients already in the waiting room wanting to see you. The pain has been present for six weeks, starting two days after moving heavy furniture at home. The pain is described as an ache (4 to 5 out of 10). There is no radiation. He has had previous, similar episodes of low-back pain that have lasted one to two weeks. The patient has no other health concerns. The patient has seen you weekly over the last four weeks for his low-back pain and complains that it is not improving. He is frustrated with his lack of improvement and thinks something different needs to be done. He is dissatisfied that he has not already been referred for further investigation, and insists that you refer him now.

* 19. Which investigations would you order, undertake or recommend for this patient at this visit? Please tick all that apply:

- [ ] Lumbosacral plain x-ray
- [ ] Full spine plain x-ray
- [ ] None

20. Please tell us why you would, or would not, order, undertake or recommend investigations

Patient Vignette 4/5

A 28 year old woman has suffered from low-back pain for a week. She has been unable to do her job managing a hospital cafeteria for this time. She walks slowly into your consultation room, holding her back and grimacing. You notice her abnormal posture. She sits with a loud groan. She says she has severe low-back pain, describing it as 9 out of 10. While anxious to return to work, she feels immobilised by the pain. There is no history of trauma. The pain is in the low-back area, without radiation. On physical examination there is marked limitation of anterior flexion and tenderness in the left paraspinal region. The neurological examination is normal with straight leg raising to 90 degrees. She has had numerous episodes of back pain in the past but thinks this is the worst episode she has ever had and is very worried that whatever is causing her problem is getting worse.

* 21. Which investigations would you order, undertake or recommend for this patient at this visit? Please tick all that apply:

- [ ] Lumbosacral plain x-ray
- [ ] Full spine plain x-ray
- [ ] None

22. Please tell us why you would, or would not, order, undertake or recommend investigations

Patient Vignette 5/5

A 67-year-old woman attends your clinic. She has low-back pain that started immediately after she fell off a chair at home 4 days ago while reaching for her glasses. The pain has been constant since then, although she gets some slight relief from paracetamol.

* 23. Which investigations would you order, undertake or recommend for this patient at this visit? Please tick all that apply:

- ☐ Lumbosacral plain x-ray
- ☐ Full spine plain x-ray
- ☐ None

24. Please tell us why you would, or would not, order, undertake or recommend investigations

Further comments

25. If you wish to add any comments about the survey, please include them in the box below:

To enter a draw for an iPad, please e-mail your name and phone number (Subject: iPad Draw) to Diana De Carvalho at **Diana.DeCarvalho@med.mun.ca**

Please note your draw entry will not be linked in any way to your survey responses.
