# Supplementary material for: Knowledge of and adherence to radiographic guidelines for low back pain: a survey of chiropractors in Newfoundland and Labrador, Canada
Source: Chiropr Man Therap. 2021 Jan 18;29:4. doi: 10.1186/s12998-020-00361-2 (PMC7812732; doi:10.1186/s12998-020-00361-2)
Supplement: Supplementary file 1 — Additional file 1. [file 12998_2020_361_MOESM1_ESM.zip › Supplementary Material 1_Changes from the Jenkins Survey_ESM.docx]

Adaptations to the Jenkins survey were minor and included seven changes:

[1] Refining whether respondents were currently in practice (to options for “No” were given: “have been away from practice greater than one year”, in which case these respondents were excluded from the rest of the survey, and “currently on leave less than or equal to one year”, to ensure we did not inadvertently exclude practitioners that could otherwise contribute meaningfully to the results of this study but happened to be temporarily away from practice due to maternity/medical leaves at the time of the study.

[2] Accounting for regional differences in language (replacing “metropolitan” with “urban”).

[3] To exclude questions irrelevant to the province of Newfoundland and Labrador (“Which Australian state or territory do you practice?” and “Where do you refer your patients for x-rays?” since all practitioners in this region only have x-rays taken at hospitals).

[4] To rearrange otherwise identical questions to optimize flow.

[5] To update and expand the list of potential radiographic guidelines in response to the question “Which radiographic guideline/s are you aware of? (Please mark all that apply)”.

[6] To gather additional information regarding additional training in radiographic indications or guidelines (including a comment box asking for what training as been undertaken as opposed to simply answering yes).

Finally, [7]“Please select a response that indicates how often you refer for x-rays of the lumbar spine” was removed to shorten the survey and because our research team decided it was of little help to address our primary research questions surrounding awareness and beliefs; further, responses could be biased with no way of validating how true they were.
